# Impact of *HFE *genetic testing on clinical presentation of hereditary hemochromatosis: new epidemiological data

**DOI:** 10.1186/1471-2350-6-24

**Published:** 2005-06-01

**Authors:** Virginie Scotet, Gérald Le Gac, Marie-Christine Mérour, Anne-Yvonne Mercier, Brigitte Chanu, Chandran Ka, Catherine Mura, Jean-Baptiste Nousbaum, Claude Férec

**Affiliations:** 1 INSERM U 613 "Génétique moléculaire et génétique épidémiologique", Brest, France; 2 Etablissement Français du Sang, Site de Brest, Brest, France; 3 Université de Bretagne Occidentale, Brest, France; 4 Service d'Hépato-Gastroentérologie, Centre Hospitalier Universitaire La Cavale Blanche, Brest, France

## Abstract

**Background:**

Hereditary hemochromatosis (HH) is a common inherited disorder of iron metabolism in Northern European populations. The discovery of a candidate gene in 1996 (*HFE*), and of its main mutation (C282Y), has radically altered the way to diagnose this disease. The aim of this study was to assess the impact of the *HFE *gene discovery on the clinical presentation and epidemiology of HH.

**Methods:**

We studied our cohort of 415 patients homozygous for the C282Y allele and included in a phlebotomy program in a blood centre in western Brittany, France.

**Results:**

In this cohort, 56.9% of the patients were male and 21.9% began their phlebotomy program before the implementation of the genetic test. A significant decrease in the sex ratio was noticed following implementation of this DNA test, from 3.79 to 1.03 (p < 10^-5^), meaning that the proportion of diagnosed females relatives to males greatly increased. The profile of HH patients at diagnosis changed after the DNA test became available. Serum ferritin and iron values were lower and there was a reduced frequency of clinical signs displayed at diagnosis, particularly skin pigmentation (20.1 vs. 40.4%, OR = 0.37, p < 0.001) and hepatomegaly (11.0 vs. 22.7%, OR = 0.42, p = 0.006). In contrast, fatigue became a more common symptom at diagnosis (68.0 vs. 51.2%, OR = 2.03, p = 0.004).

**Conclusion:**

This study highlights the importance of the *HFE *gene discovery, which has simplified the diagnosis of HH and modified its clinical presentation and epidemiology. This study precisely measures these changes. Enhanced diagnosis of *HFE*-related HH at an early stage and implementation of phlebotomy treatment are anticipated to maintain normal life expectancy for these patients.

## Background

Hereditary hemochromatosis (HH) is a common genetic disorder of iron metabolism that is usually inherited in an autosomal recessive pattern and associated with missense mutations in the *HFE *gene. This pathology displays a large genetic heterogeneity because several other types of hemochromatosis, associated with different genes and patterns of inheritance, have been reported [1]: HH type 2B is a juvenile form linked to the *HAMP *gene (encoding for hepcidin) [2-4], HH type 3 is linked to the *TfR2 *gene (encoding for transferrin receptor 2) [5-7], HH type 4 is linked to the *SLC11A3 *gene (encoding for ferroportin, an intestinal iron transporter) [8,9]. and HH type 5 is linked to a gene encoding subunit H of ferritin [10]. Moreover, the gene responsible for juvenile hemochromatosis (HH type 2A), and whose protein product is called hemojuvelin, has recently been cloned [11].

The main form of HH (i.e. type I which is linked to the *HFE *gene) occurs predominantly in Northern European populations, with a prevalence of approximately 3 to 8 in 1000 [12-16]. It is characterised by excessive iron absorption, which progressively leads to the destruction of tissues in different organs of the body. After a phase of latency, the first signs of biochemical expression appear, generally around the age of 20. This is characterised by increases in serum iron parameters (transferrin saturation, ferritin). The clinical expression manifests later during adulthood, generally around the age of 40 in males and later in females, around the age of 50, because of the protective effects of menstrual blood loss and pregnancies [17-19]. The clinical picture may include at an early stage, non-specific symptoms such as persistent fatigue and arthralgias, and at a later stage, clinical signs such as skin pigmentation, hepatomegaly, arthropathy, cardiomyopathy, diabetes and cirrhosis [20-22]. Classically, this clinical expression occurs more frequently in males than in females (sex ratio of 3:1) [23].

HH can be treated or prevented by periodic phlebotomies. This simple and efficient treatment prevents iron accumulation and clinical complications. Without this early treatment, the disease may progress towards irreversible damage such as cirrhosis and hepatocellular carcinoma [17-19].

A candidate gene for HH type 1, *HFE*, was identified in 1996 on chromosome 6 and encodes the HFE protein, a transmembrane glycoprotein that is implicated in modulation of iron uptake [24,25]. Currently, about twenty different mutations have been identified in this gene worldwide and, one of them, termed C282Y, is present at homozygous state in 80 to 95% of HH patients. This molecular anomaly corresponds to the substitution of a tyrosine for a cysteine at amino acid 282, which prevents formation of a disulfide bond [24,26]. The two other most common mutations of the *HFE *gene are associated with milder forms of HH (H63D and S65C) [1,21,27-29].

The discovery of the *HFE *gene in 1996 and the fact that one of its mutations (C282Y) is responsible for the large majority of HH cases enabled the implementation of efficient strategies for molecular diagnosis [30], what has altered the way in which HH is diagnosed. Initially, the diagnosis relied on a high index of suspicion associated with evidence of elevated iron parameter values [18,20,22]. Following discovery of the *HFE *gene, a DNA test was proposed to confirm the diagnosis of HH. Such a test, which allows the detection of at least the C282Y mutation, is now widely available. This discovery has simplified the diagnostic strategy and enabled pre-symptomatic or earlier diagnosis in some patients. If phlebotomy treatment is implemented before the appearance of irreversible damage, the excess iron can be removed and patients have a normal life expectancy [20,31].

In this study, we assessed the impact of *HFE *genetic testing on the clinical presentation and epidemiology of HH in a cohort of 415 patients homozygous for the C282Y mutation who were followed in a blood centre in western Brittany, France. This report contains objective data to measure this impact.

## Methods

### Study population

The present study was conducted in Brittany, a region of nearly three million inhabitants located in the north-western part of France, where HH is particularly frequent (carrier rate: 1 in 7) [32]. This disease presents a large genetic and allelic heterogeneity, but as the majority of patients are homozygous for the C282Y mutation, we decided to include in this study only the patients homozygous for this mutation. This study included all the C282Y homozygous patients who are or were included in a phlebotomy program in a blood centre of western Brittany and who presented with transferrin saturations of greater than or equal to 45%. The first patients began their treatment in the early eighties and the last date of patient entry for inclusion in this study was December 31^st ^2003. The proportion of homozygous C282Y patients before and after the implementation of the genetic test did not change significantly.

### Clinical questionnaire

A detailed clinical questionnaire was completed during the clinical exam performed at the first visit of patients to the blood centre (i.e. at entry in the phlebotomy program). Information contained in this questionnaire was previously described in detail [33]. Briefly, it provided information regarding socio-demographic characteristics of patients, their age at diagnosis, the circumstances of HH discovery, the biochemical parameters and the clinical signs associated at the time of diagnosis. This questionnaire also included data related to the treatment, such as the number and quantity of phlebotomies needed to reach depletion and the quantity of iron extracted. The date of the beginning of the treatment (*i.e. *entry into the phlebotomy program) was also documented. This date was used to determine if the treatment of patients began before or after the availability of the genetic test (*i.e. *1996). The intake of alcohol was assessed by a detailed item included in the questionnaire, which measured the number of glasses of alcohol drunk each day (including glasses of wine, beer and liquor). These data enabled the quantity of ethanol (in grams) consumed each day, by each patient of the cohort, to be determined. Excessive alcohol consumption was defined as a daily consumption greater than or equal to 60 grams of ethanol [33].

### Determination of biochemical parameter levels and of *HFE *genotype

Serum iron parameters (*i.e. *serum iron, serum ferritin and serum transferrin saturation) were determined by standard biochemical methods (including collection of serum after a 12-hour fast, confirmation by at least two measurements).

Analysis of the C282Y mutation relies on amplification of the specific gene region by the polymerase chain reaction, followed by mutation detection using restriction enzymes [29,32]. Recently, another method was adopted for this analysis: the denaturing high-performance liquid chromatography method [34]. If the DNA test confirms the presence of mutations in a patient, family testing is offered to the relatives of this newly diagnosed patient. Family testing combines the collection of biochemical and clinical evidence of iron overload for the patients relatives with analysis of the main *HFE *mutations. Before 1996, family testing was already possible by analysis of HLA haplotypes in families [35] and this was commonly practised in our region where the disease incidence is high. The genotype of the patients diagnosed before 1996 was retrospectively determined when the genetic test became available.

### Statistical analysis

Data were analysed using Epi-Info software (version 6.04; Centers for Disease Control and Prevention, Atlanta, Georgia) and SAS statistical package (version 8.2; SAS Institute). Quantitative variables, expressed as means and standard deviations, were compared with the Student's *t *test or ANOVA, whereas qualitative variables, expressed as percentages, were compared with the Chi square test or the Fisher's exact test (in case of small sample size). A logarithmic transformation was performed for the serum ferritin variable which had a skewed distribution. A significance level of 5% was used for all of the analyses which were performed two-sided.

The analyses consisted of determining the influence of the implementation of the genetic test on the sex ratio, the age at diagnosis, the circumstances of HH discovery, the levels of biochemical parameters and the frequency of clinical signs associated at the time of diagnosis. The comparisons of biochemical and clinical data, which were made separately for males and females, were adjusted for age at diagnosis and for alcohol consumption, because we showed previously that excessive alcohol consumption (>60 g/day) increased HH expressivity [33]. The relation between the time of diagnosis (i.e. before or after availability of *HFE *genotyping) and each of the different clinical symptoms was assessed by calculating the odds-ratio (OR) and its 95% confidence interval (CI).

This study complied with French bioethical regulations. Informed consent of patients was obtained before blood samples were taken.

## Results

### Description of the study population

This study included 415 C282Y homozygous patients of whom 56.9% were male, resulting in a sex ratio of 1.3:1 (236/179). The age at diagnosis, ranging from 13 to 76 years, was significantly higher in females than in males (48.5 yrs (σ = 14.3) vs. 46.1 yrs (σ = 12.5), p = 0.037). Overall, the diagnosis was mainly made on the basis of clinical features (61.4%) or through family testing (30.9%). The circumstances of diagnosis remained unknown for one patient. Among these 415 patients, 21.9% began their phlebotomy program before the implementation of the molecular testing (n = 91, 72 males and 19 females) and 78.1% after this date (n = 324, 164 males and 160 females).

### Characteristics of patients before and after the implementation of the genetic test

#### Socio-demographic characteristics of patients in relation to the type of diagnosis (based on *HFE *genotyping or not)

**Figure 1:**
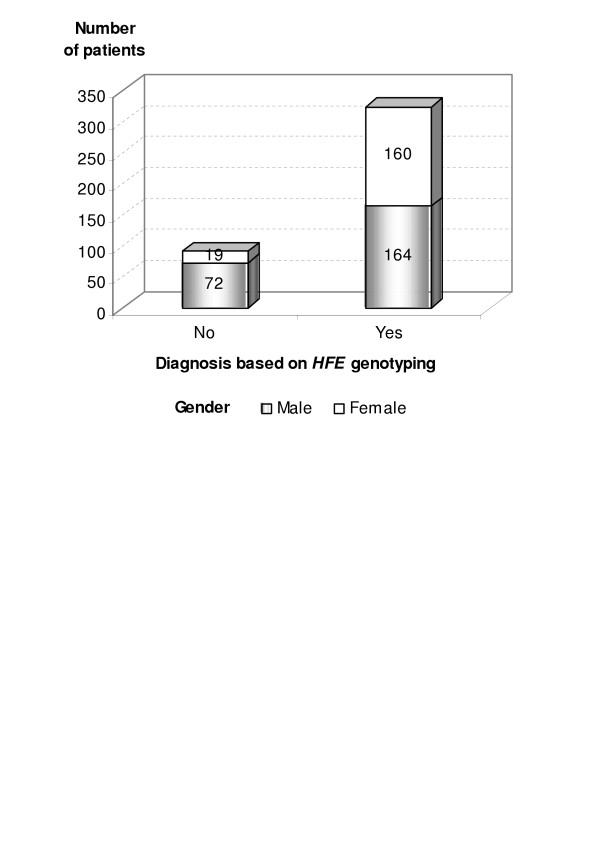
Evolution of the sex ratio: number of males and females diagnosed before and after the implementation of the genetic test.

A significant decrease in the sex ratio was noted following development of the genetic test, as illustrated in figure 1. The sex ratio was 3.79 (72/19) before discovery of the gene and 1.03 (164/160) after this date (p < 10^-5^), meaning that the proportion of females diagnosed since 1996 increased greatly: from 20.9% (19/91) to 49.4% (160/324). These females seemed to be diagnosed earlier. Their age at diagnosis tended to decrease from 52.9 yrs (σ = 9.8) before the implementation of the genetic test to 47.9 yrs (σ = 14.7) after this date. Nevertheless, this difference was not significant because a small number of women was diagnosed before 1996 (n = 19, p = 0.210). This pattern was not observed in males, their age at diagnosis increased from 42.7 yrs (σ = 10.4) to 47.6 yrs (σ = 13.1) after 1996 (p = 0.007).

After introduction of molecular testing, the symptom of unexplained and persistent fatigue was more commonly present at diagnosis of HH. The frequency of this symptom increased from 51.2 to 68.0% following introduction of the DNA-based testing (OR = 2.03, 95% CI: 1.21, 3.41; p = 0.004), notably in males (from 47.1 to 58.4%). On the other hand, the proportion of patients detected by family testing was not significantly changed after 1996 (31.3 vs. 29.7%).

#### Biochemical parameters in relation to the type of diagnosis (based on *HFE *genotyping or not)

Differences were also observed in biochemical parameter values. The results of the comparison of the biochemical data of patients before and after the development of the genetic test are presented in table 1. Serum ferritin and serum iron values were significantly lower in patients diagnosed after implementation of the genetic test (serum ferritin: 810.4 vs. 1788.8 μg/liter, p < 0.001; serum iron: 34.9 vs. 39.8 μmol/liter, p < 0.001). These results were observed in males (serum ferritin: 1136.9 vs. 1886.6 μg/liter, p < 0.001; serum iron: 36.7 vs. 40.7 μmol/liter, p < 0.001) and in females (serum ferritin: 475.7 vs. 1418.0 μg/liter, p < 0.001; serum iron: 33.1 vs. 36.6 μmol/liter, p = 0.052). As shown in table 1, the results remained unchanged after adjustment for age at diagnosis and alcohol consumption. In this cohort, 8.0% of the patients declared having excessive alcohol consumption (≥ 60 g/day; n = 33) comprising 9.9% of the patients diagnosed before 1996 (n = 9) and 7.4% of those diagnosed after 1996 (n = 24). A decrease was not observed for the third iron parameter, transferrin saturation (79.5 vs. 79.4%, p = 0.930), for which an elevated value (>45%) was used as a selection criterion for this study.

#### Clinical signs in relation to type of diagnosis (based on *HFE *genotyping or not)

**Figure 2:**
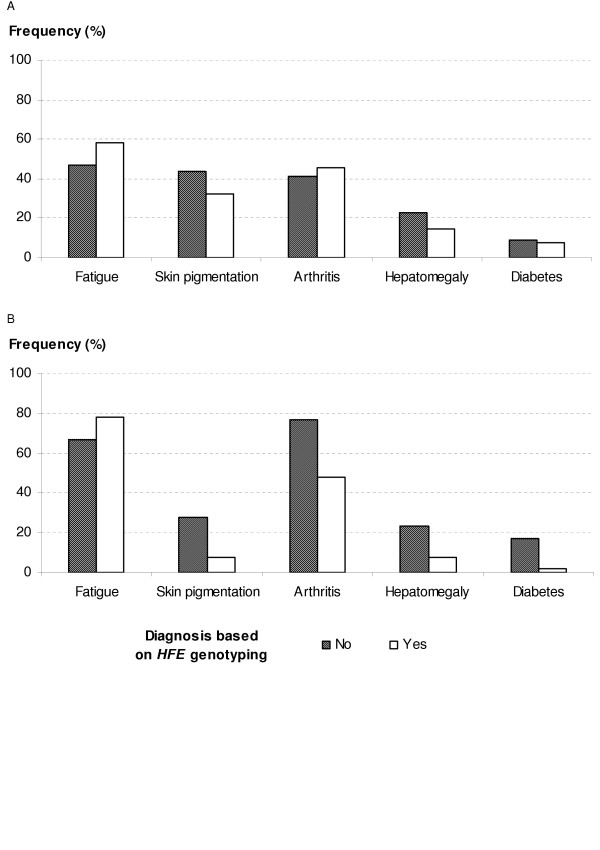
**(A)** Frequency of the main clinical signs and symptoms at the time of diagnosis in males, before and after the implementation of the genetic test. **(B)** Frequency of the main clinical signs and symptoms at the time of diagnosis in females, before and after the implementation of the genetic test

The frequency of the main clinical signs observed at the time of diagnosis before and after the availability of the molecular testing is shown in figures 2A and 2B. The clinical signs were less frequent in the subjects diagnosed after the development of the genetic test, particularly skin pigmentation (20.1 vs. 40.4%; OR = 0.37, 95% CI = 0.22, 0.63; p < 0.001) and hepatomegaly (11.0 vs. 22.7%; OR = 0.42, 95% CI = 0.21, 0.83; p = 0.006). This change was more significant in women. Indeed, only the two signs mentioned above tended to be less frequently observed in men following the introduction of genetic testing although these changes did not reached statistical significance (skin pigmentation: 32.1 vs. 43.7%; OR = 0.61, 95% CI = 0.33, 1.13; p = 0.089 – hepatomegaly: 14.7 vs. 22.5%; OR = 0.59, 95% CI = 0.27, 1.32; p = 0.160), whereas in women, four symptoms were significantly less frequent following implementation of molecular testing: skin pigmentation (7.6 vs. 27.8%; OR = 0.22, 95% CI = 0.06, 0.84; p = 0.006), hepatomegaly (7.3 vs. 23.5%; OR = 0.26, 95% CI = 0.06, 0.86; p = 0.028), arthritis (47.5 vs. 76.5%; OR = 0.28, 95% CI = 0.07, 0.98; p = 0.023) and diabetes (2.0 vs. 16.7%; OR = 0.10, 95% CI = 0.01, 0.71; p = 0.001). These results were similar in the sub-group of patients having no excessive alcohol consumption (data not shown).

## Discussion

The discovery of the *HFE *gene in 1996 constituted a considerable advance in the medical and scientific field. This discovery concerned one of the most common inherited disorders in white populations, HH – a disorder that was complex to diagnose but for which a treatment existed – and identified one of the few undiscovered genes that has an important impact on public health.

The symptomatology of HH has evolved over the past years and it is now rare to diagnose severe forms of the disease, associated with diabetes, cirrhosis and darkened skin [36,37]. Through a survival analysis based on a cohort of 251 patients diagnosed between 1947 and 1991, Niederau *et al. *showed that the percentage of patients with early diagnoses increased 3-fold during the period of 1970–1981 compared to the period of 1947–1969, and that there was a further 20–25% increase in the early diagnosis rate during the period of 1981–1991 [37]. These changes occurred before the discovery of the *HFE *gene, and were probably the consequences of improved education of physicians and the implementation of HLA testing for family members of probands.

The current study highlights the importance of the discovery of the *HFE *gene in 1996 and demonstrates how the clinical presentation and epidemiology of HH have changed since the availability of the DNA test. Our results objectively measure these changes, and show that the sex ratio of this disease has altered: the proportion of females currently diagnosed has increased and has reached that of males. This study also highlights that the profile of HH patients has changed: the patients have lower iron parameter values (serum ferritin and iron) and a lower frequency of clinical signs at the time of diagnosis, notably skin pigmentation and hepatomegaly. This change is more pronounced in females in whom clinical manifestations of HH appears later than in males (around the age of 50 versus around the age of 40 in males). This study included all the C282Y homozygous patients who are or were included in a phlebotomy program in a blood centre of western Brittany. For the patients diagnosed before the implementation of the genetic test, the genotype was retrospectively determined in 1996 if they were still alive at this date. Consequently, the patients who died before 1996 were not genotyped and not included in this study. With this bias, some severe cases of the disease have been missed and the difference between the two groups should therefore be even greater than reported here.

Identification of the *HFE *gene and of its main mutation (C282Y) has greatly simplified diagnosis of, and family testing for, HH [20,31]. The fact that homozygosity for the C282Y mutation is responsible for the majority of HH cases has enabled use of the molecular test for this mutation as a diagnostic criterion for HH. Before the genetic test was available, diagnosis of HH required a high index of suspicion (as the clinical signs are non-specific) and evidence of elevated iron parameters [18,20,22]. Traditionally, diagnosis was based on the measurement of transferrin saturation. A liver biopsy then enabled confirmation of iron overload by detection of elevated hepatic iron levels [27]. The discovery of the *HFE *gene enabled molecular analysis to be included in the diagnostic strategy and thus genetic testing for confirmation of the diagnosis was proposed. In this way, HH could easily be differentiated from all other types of iron overload. Currently, the diagnosis combines molecular testing with traditional biochemical methods. The diagnostic strategy is as follows: 1) To suspect the diagnosis from non-specific symptoms (such as persistent fatigue, arthralgias), and not only when presented with classical signs of HH (such as skin pigmentation, diabetes and cirrhosis); 2) Once the disease is suspected, the second step is to determine the serum transferrin saturation; 3) If the value of this iron parameter is elevated, molecular analysis of the main *HFE *mutations (C282Y +/- H63D) must be done to confirm the diagnosis of HH [20]. The diagnostic strategy has changed, and as a consequence, patients with increased iron parameter values and a genotype of HH are now diagnosed as having HH. The molecular basis of the disease has been evidenced and inclusion of genetics in the diagnostic strategy has enabled detection of iron overload that is expressed only at a biochemical level.

The *HFE *gene discovery has improved our knowledge of this complex disease. It has enabled the genotypes of patients to be determined, and by considering this information in relation to other factors such as age, gender and environment, elucidation of genotype/phenotype correlations has begun [33,38,39]. The *HFE *gene discovery also raised the complex issue of the penetrance, which is clearly incomplete [22,40-44]. It is probable that some of the patients who exhibit biochemical evidence of iron overload and a genotype of HH would never progress towards the clinical manifestations of HH. Two studies reported that less than 1% of the C282Y homozygous subjects develops clinical hemochromatosis [40,45]. Unfortunately, studies on penetrance generally suffer from bias that results in under or over-estimation of the frequency of the disease [22,46]. Until more data are available on the penetrance of the C282Y homozygous state, screening using *HFE *genotyping remains controversial [27,47].

Nevertheless, looking beyond this complex issue of penetrance, the gene discovery has led to a better understanding of some of the phenotypic variability observed in HH. This improved knowledge has been conducive to better medical education of physicians, such that they now may more often suspect a diagnosis of HH when presented with non-specific symptoms (such as unexplained and persistent fatigue) than they did previously. This education is certainly not perfect at this time but we can observe in the present study that the proportion of patients, particularly males, diagnosed with the symptom of fatigue has already increased since the availability of *HFE *genotyping. With astute clinical assessment and *HFE *genetic testing, patients can be diagnosed and treated before the appearance of irreversible damage, and this therefore avoids development of severe forms of HH. In our study, an increase in the age at diagnosis after the introduction of the DNA test was observed in men. This could be explained by the fact that a diagnosis of HH has been done in some men older than 65 presenting with fatigue, arthralgia and a discrete ferritin elevation. Those C282Y homozygous patients would probably never have been diagnosed ten years ago. The inclusion of these old diagnosed C282Y homozygous men in our cohort significantly increased the age at diagnosis of HH during the last years.

Family testing performed among the relatives of a newly diagnosed patient also enables detection of subjects in the pre-symptomatic phase. The discovery of the *HFE *gene has not significantly altered family testing for HH because, prior to 1996 it was already possible to analyze the transmission of HLA haplotypes in families (the *HFE *gene is located near the HLA complex). Such testing was commonly practiced in our region where HH is common. In our study, the proportion of patients detected by family testing before and after the introduction of *HFE *genotyping was similar (29.7% versus 31.3%). Consequently, the inclusion, in the present study, of patients identified through family testing did not alter our findings. The impact of the *HFE *gene test on the identification of HH through family testing is expected to be higher in other regions where family testing was not practiced as systematically as in our region prior to 1996.

This pre-symptomatic or early diagnosis of HH and follow-up phlebotomy treatment should prove efficacious in preventing organ damage and therefore aid in achieving normal life expectancy for patients [48]. Early detection can completely prevent premature death caused by HH. Illustrating this point, Milman *et al. *found, through analysis of a cohort of patients diagnosed in Denmark between 1945 and 1985, that the survival of HH patients without cirrhosis or diabetes mellitus was similar to that in the general population [48].

## Conclusion

In conclusion, *HFE *mutation testing has supplemented the determination of serum iron parameters as a criterion for the diagnosis of HH. This has increased the proportion of women diagnosed with HH and has decreased the frequency of certain clinical signs at diagnosis. The method of diagnosis of HH has changed and this has contributed to modify the epidemiology of this disease, with the sex ratio reduced to close to 1.0 and a weaker clinical expression than observed previously. This study highlights an example of the progress enabled by Genomic Medicine [49] and shows that knowledge of the molecular basis of a disease (following identification of its gene and mutations involved) can lead to a change in the epidemiology of that disease.

## Competing interests

The author(s) declare that they have no competing interests.

## Authors' contributions

VS contributed to the conception and design of the work, analysed the data and wrote the paper. GLG and CM were involved in genetic analysis and revised the paper. MCM, AYM, BC and JBN helped in the acquisition of data. CF contributed to the conception and design of the work and supervised the study. All authors read and approved the final manuscript.

## Pre-publication history

The pre-publication history for this paper can be accessed here:



## Figures and Tables

**Table 1:** Biochemical characteristics of the C282Y homozygous patients before and after the implementation of the genetic test.

	**Diagnosis based on *HFE *genotyping**				**Crude *p*-value**	Adjusted* *p*-value
			
	**No**		**Yes**			
	mean	(SD)	mean	(SD)		
No. of patients	91		324			
Gender (no. and % of males)	72	(79.1%)	164	(50.6%)	p < 10^-5^	-
						
**Males**						
Serum ferritin (μg/L)	1886.6	(1769.7)	1136.9	(951.0)	<0.001	<0.001
Serum iron (μmol/L)	40.7	(6.9)	36.7	(6.7)	<0.001	<0.001
Transferrin saturation (%)	79.9	(11.9)	83.0	(13.2)	0.090	0.134
						
**Females**						
Serum ferritin (μg/L)	1418.0	(1457.9)	475.7	(744.6)	<0.001	<0.001
Serum iron (μmol/L)	36.6	(8.2)	33.1	(7.4)	0.052	0.029
Transferrin saturation (%)	77.2	(13.4)	75.9	(14.4)	0.710	0.714

* Adjusted for age at diagnosis and alcohol consumption
